# Value of computed tomography Hounsfield units in predicting pedicle screw loosening in the thoracic spine

**DOI:** 10.1038/s41598-022-23142-8

**Published:** 2022-10-31

**Authors:** Minsu Lee, Eugene Lee, Joon Woo Lee

**Affiliations:** 1 Department of Radiology, Seoul National University Bundang Hospital, 82, Gumiro 173beon-gil, Bundang-gu, Seongnam-si, Gyeonggi-do 13620 Republic of Korea; 2 Department of Radiology, Seoul National University College of Medicine, 103, Daehak-ro, Jongno-gu, Seoul, 03080 Republic of Korea

**Keywords:** Diseases, Medical research, Risk factors, Signs and symptoms

## Abstract

We evaluated the feasibility of using the Hounsfield unit (HU) value of the vertebral body to predict screw loosening in the thoracic spine. Consecutive patients who underwent thoracic spinal fusion surgery (from 2014 to 2020) were retrospectively identified. Patients with pedicle screw loosening in the upper instrumented vertebra (UIV) on postoperative computed tomography were included in the “loosening” group. The control group comprised an equal number of age-, sex-, and UIV-matched patients without screw loosening. Preoperative HU values at the UIV and lumbar T-scores were compared between the groups; receiver operating characteristic curves were constructed for HU values and T-scores to predict screw loosening, and the best cutoff values were determined. The same statistical analyses were performed for each subgroup, i.e., upper (T1–T4) and lower (T9–T12) thoracic levels. Forty-six patients each were included in the loosening and control groups. A significant between-group difference of HU values was noted for the lower thoracic UIV (loosening = 99.3, control = 126.3; *p* = 0.02) but not for the upper thoracic UIV (loosening = 171.8, control = 146.0, *p* = 0.70). T-scores did not differ between the groups for the lower (*p* = 0.14) and upper (*p* = 0.56) thoracic UIV. For the lower thoracic UIV, the area under the receiver operating characteristic curve was 0.660 for HUs (*p* = 0.01; 95% confidence interval [CI] 0.541–0.766) and 0.601 (*p* = 0.13; 95% CI 0.480–0.713) for T-scores. The optimal cutoff value for HUs was 126.3. Using this cutoff, HU values showed a better positive predictive value, negative predictive value, and accuracy compared to T-scores in predicting screw loosening.

## Introduction

The prevalence of degenerative spinal disease is increasing with the increase in life expectancy. Previous studies have shown an important association between aging and adult spinal deformities^1,2^. Conservative treatment, such as exercise or physiotherapy, is recommended as a first-line treatment, but if ineffective, surgical intervention is considered. With the growing prevalence of adult spinal deformity, the number of surgical corrections has recently increased. Spinal fusion with pedicle screw fixation is generally the preferred surgical procedure, and depending on the severity, the surgery can be extended to the thoracic spine. Owing to advances in surgical techniques and implants, the outcomes of spinal deformity surgeries have considerably improved in the past decade^3^. However, screw loosening is still a clinically important complication of pedicle screw fixation, because it can lead to fusion failure and pseudoarthrosis^4^. Screw loosening is frequently encountered in the thoracic spine, especially at the upper instrumented vertebra (UIV), causing proximal junctional kyphosis.

Vertebral bone quality, assessed as a form of bone mineral density (BMD), is the most commonly reported risk factor for pedicle screw loosening^5,6^. Since osteoporosis is frequently encountered in patients requiring spinal surgery, preoperative evaluation of BMD is recommended for surgical planning and predicting clinical outcome^7^. Dual-energy X-ray absorptiometry (DEXA) is the most widely used parameter of bone quality. However, DEXA might overestimate the BMD of the lumbar spine and miss osteoporosis in patients with spinal degeneration or aortic calcifications due to its projectional nature^8,9^. Moreover, results from DEXA in the spine are only reliable from the L1–L4 vertebrae and are not commonly accepted as a standard modality in the thoracic spine field^10^.

In previous studies, Hounsfield unit (HU) values of the vertebral body obtained via computed tomography (CT) demonstrated good correlation with DEXA results^11,12^. In the lumbar region, attempts have been made to predict pedicle screw loosening using HU values on CT instead of BMD measured via DEXA^13,14^. However, it is unclear whether the HU value of the vertebral body is useful in predicting loosening in the thoracic vertebra.

The purpose of this study was to evaluate the feasibility of using the HU value of the vertebral body to predict screw loosening in the thoracic spine.

## Materials and methods

### Patient selection

**Figure 1:**
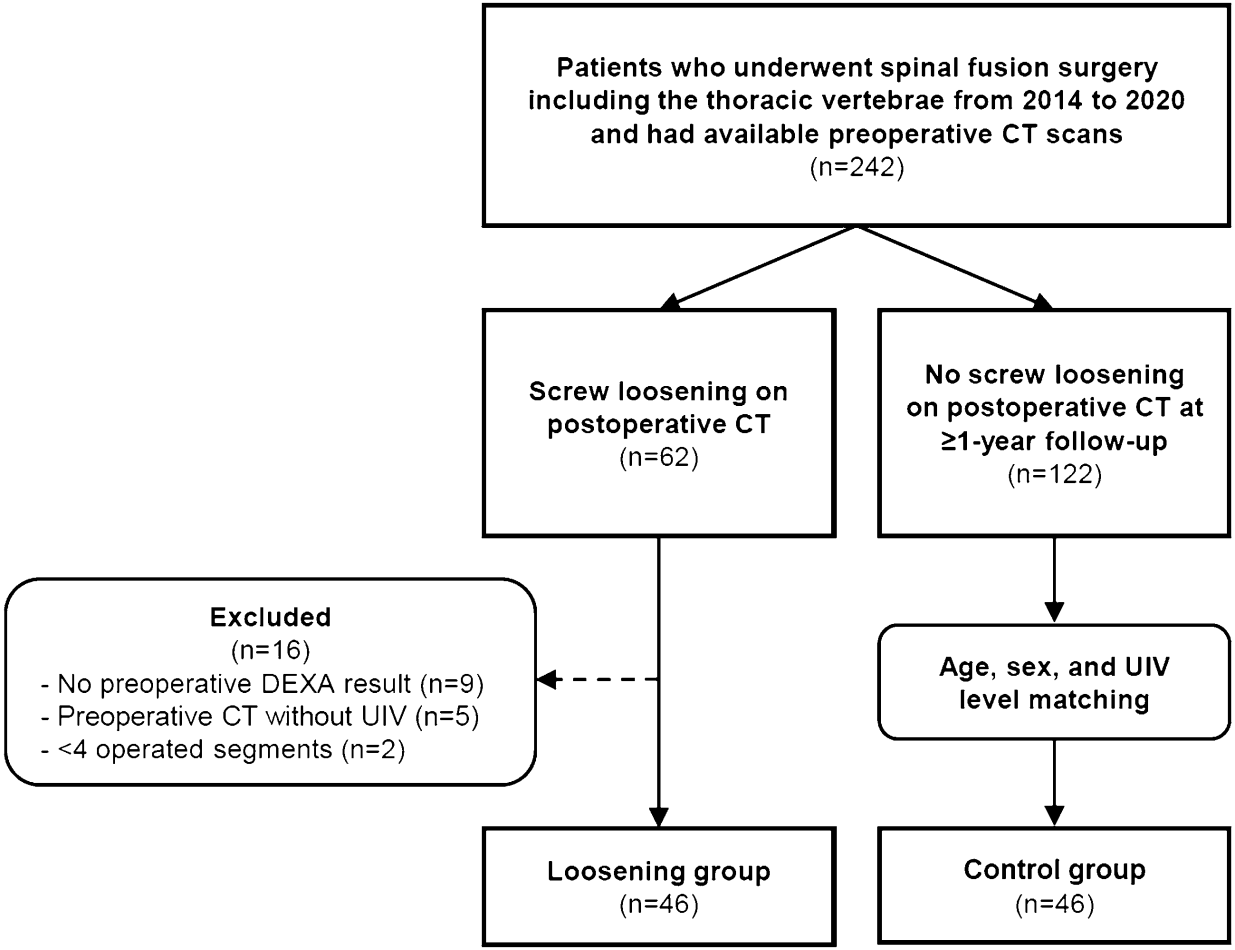
Flow diagram of patient selection. DEXA, dual-energy X-ray absorptiometry; UIV, upper instrumented vertebra.

This retrospective study was approved by the Institutional Review Board of Seoul National University Bundang Hospital, and the requirement for informed consent was waived because of the retrospective nature of the study. All patient data were analyzed anonymously in accordance with relevant guidelines and regulations. We reviewed the electronic medical records of our institution to identify 242 consecutive patients who underwent spinal fusion surgery between January 2014 and December 2020 and met the following inclusion criteria: (1) spinal fusion surgery including the thoracic vertebra and (2) preoperative CT data available. Among them, 62 patients who had been diagnosed with screw loosening at their upper instrumented level on postoperative CT were included in the “loosening” group. Sixteen patients were excluded from the loosening group for the following reasons: lack of preoperative DEXA results (n = 9), preoperative CT without including the UIV (n = 5), and < 4 operated segments (n = 2). As a result, 46 patients (37 women and 9 men) were finally included in the loosening group. Among the 242 patients who met the inclusion criteria, 122 patients without screw loosening on CT during a postoperative follow-up of ≥ 1 year were included in the control group. Patients included in the control group were selected using a one-to-one matching of age, sex, and UIV level. Finally, 46 patients were included in the control group. The selection process is illustrated in Fig. 1. Demographic variables, surgical levels, and preoperative DEXA results were recorded in both groups.

### Image analysis and Hounsfield unit measurement

All CT scans were performed using multidetector-row helical CT scanners (Somatom Definition Edge, Siemens, Munich, Germany; Mx 8000 IDT 16, Philips Medical Systems, Best, Netherlands; Brilliance 64, Philips Medical Systems; iCT 256, Philips Medical Systems; IQon Spectral CT, Philips Medical Systems). The CT parameters comprised a peak potential of 120 kVp, slice thickness of 3 mm, and increments of 2 or 3 mm. The mA-second setting ranged from 167 to 315 mAs.

**Figure 2:**
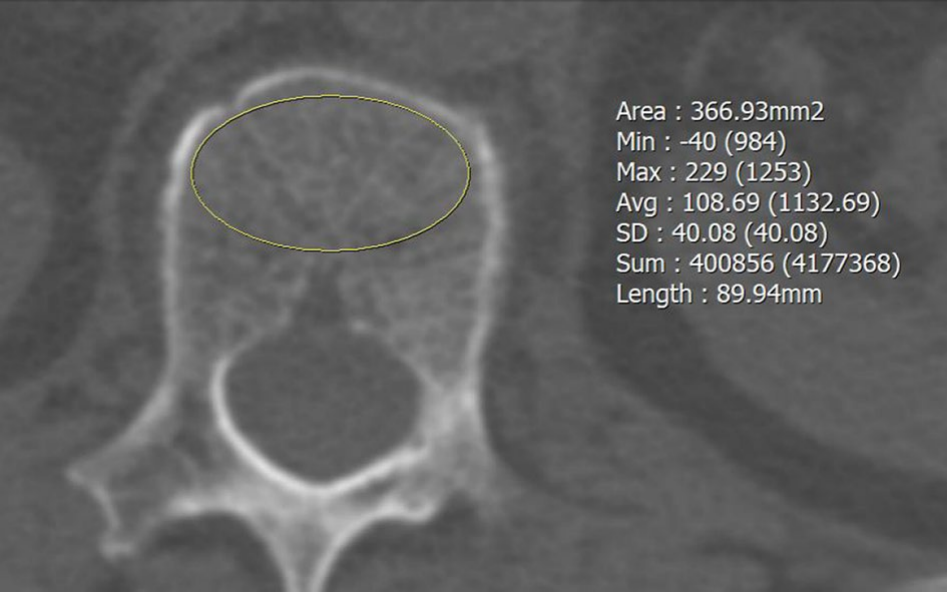
Example of CT Hounsfield unit (HU) measurement in an axial image of an upper instrumented vertebra at the mid-body level. The mean HU value was automatically derived by the picture archiving and communication system. *Min* minimum, *max* maximum, *avg* average, *SD* standard deviation.

A board-certified radiologist (with 1 year of experience in musculoskeletal radiology) who was unaware of the DEXA results independently assessed the HU values on preoperative CT in the loosening and control groups using the picture archiving and communication system (Infinitt PACS, Infinitt Healthcare, Seoul, South Korea). HU values were measured using previously published methods^11,14^. The largest elliptical regions of interest were drawn on the axial images at the middle level of the UIV body, avoiding the cortical edges and the basivertebral vein. The mean HU value of the regions of interest was calculated automatically using the PACS as shown in Fig. 2. All postoperative CT scans were evaluated by either of two experienced spine radiologists (18 and 9 years of experience in musculoskeletal radiology, respectively). Pedicle screw loosening was defined as a radiolucent area around the screw that exceeded 1 mm on the follow-up CT scan.

All DEXA scans were performed with a GE Healthcare (Chicago, IL, USA) Lunar Prodigy densitometer and a Hologic Horizon DEXA system (Marlborough, MA, USA). BMD was measured for the femur (neck, trochanter, and total femur) and the L1–L4 vertebrae, and the T-score was calculated for each. Among the DEXA results, the lowest T-score of the lumbar vertebrae was used as a representative value.

### Statistical analysis

Continuous variables are presented as means with their standard deviations, and categorical variables are presented as percentages. The HU values of the loosening and control groups were compared using the Mann–Whitney U test. Receiver operating characteristic curves were constructed for HU values and DEXA for the prediction of screw loosening, and the best cut-off values were determined. The areas under the two curves (AUCs) were compared using the DeLong test. Using the cut-off value, the sensitivity, specificity, positive predictive value (PPV), negative predictive value (NPV), and accuracy of HU values and DEXA results were investigated for the ability to predict screw loosening. All statistical analyses were performed for all subjects and for each subgroup according to the UIV level. Statistical calculations were performed using MedCalc (version 20; MedCalc Software, Ostend, Belgium). Statistical significance was set at *p* < 0.05.

## Results

### Patient characteristics

The characteristics of the patients in the loosening (n = 46) and control (n = 46) groups are summarized in Table 1. The loosening group included 9 men and 37 women (mean age 70.3 ± 8.2 years). The control group included 7 men and 39 women (mean age 70.0 ± 7.6 years). The mean follow-up period was 335.4 ± 165.8 days for the loosening group and 797.2 ± 346.1 days for the control group. The mean HU value was 115.1 ± 62.8 for the loosening group and 129.7 ± 55.7 for the control group. The lowest T-score of the lumbar spine measured with DEXA was − 1.7 ± 1.6 in the loosening group and − 1.4 ± 1.6 in the control group. The two groups were identical in the proportions of osteopenia (12 of 46 patients, 26.1%) and osteoporosis (21 of 46 patients, 45.6%). In the loosening group, UIVs were located at the upper thoracic level (T1–T4) in 10 patients (21.7%) and at the lower thoracic level (T9–T12) in 36 patients (78.3%). In the control group, UIVs were located at the upper thoracic level (T1–T4) in 8 patients (17.4%) and at the lower thoracic level (T9–T12) in 38 patients (82.6%).

**Table 1:** Characteristics of the loosening and control groups.

Variables	Loosening group (n = 46)	Control group (n = 46)
Age (years)^†^	70.3 ± 8.2	70.0 ± 7.6
**Sex**		
Male	9 (19.6)	7 (15.2)
Female	37 (80.4)	39 (84.8)
**Upper instrumented level**		
Upper thoracic vertebra (T1-T4)	10 (21.7)	8 (17.4)
Lower thoracic vertebra (T9-T12)	36 (78.3)	38 (82.6)
Follow-up period^†^	335.4 ± 165.8	797.2 ± 346.1
Mean Hounsfield unit value^†^	115.1 ± 62.8	129.7 ± 55.7
Lowest T-score of L-spine^†^	-1.7 ± 1.6	-1.4 ± 1.6
Normal	13 (28.3)	13 (28.3)
Osteopenia	12 (26.1)	12 (26.1)
Osteoporosis	21 (45.6)	21 (45.6)

Unless otherwise indicated, data are presented as numbers of patients (%).

^†^Data are presented as mean ± standard deviation.

### Comparison of Hounsfield unit values and T-scores

The mean HU value of the loosening group was lower than that of the control group, but the difference was statistically nonsignificant (115.1 vs. 129.8, *p* = 0.07). In patients with UIVs at the lower thoracic level, the loosening group had significantly lower HU values than the control group (99.3 vs. 126.3, *p* = 0.02) (Table 2). In patients with UIVs at the upper thoracic level, higher HU values were observed in the loosening group than in the control group, but there was no significant difference (171.8 vs. 146.0, *p* = 0.70). The mean T-score was also lower in the loosening group than in the control group, but no statistical significance was identified (− 1.7 vs. − 1.4, *p* = 0.35). The T-score failed to demonstrate a difference between the loosening and control groups in patients with UIVs at the upper thoracic level (− 1.3 vs. − 1.8, *p* = 0.56) and lower thoracic level (− 1.3 vs. − 1.8, *p* = 0.14).

**Table 2:** Comparison of HU values and T-scores according to UIV level.

Variables	Loosening group	Control group	*p*-value
Mean HU value	115.1 ± 62.8	129.7 ± 55.7	0.07
Upper thoracic UIV	171.8 ± 92.3	146.0 ± 71.2	0.70
Lower thoracic UIV	99.3 ± 41.3	126.3 ± 52.4	0.02
Mean lowest lumbar T-score	− 1.7 ± 1.6	− 1.4 ± 1.6	0.35
Upper thoracic UIV	− 1.3 ± 2.1	− 1.8 ± 1.5	0.56
Lower thoracic UIV	− 1.8 ± 1.4	− 1.3 ± 1.6	0.47

*HU* Hounsfield unit, *UIV* upper instrumented vertebra.

### Receiver operating characteristic curve analysis

**Figure 3:**
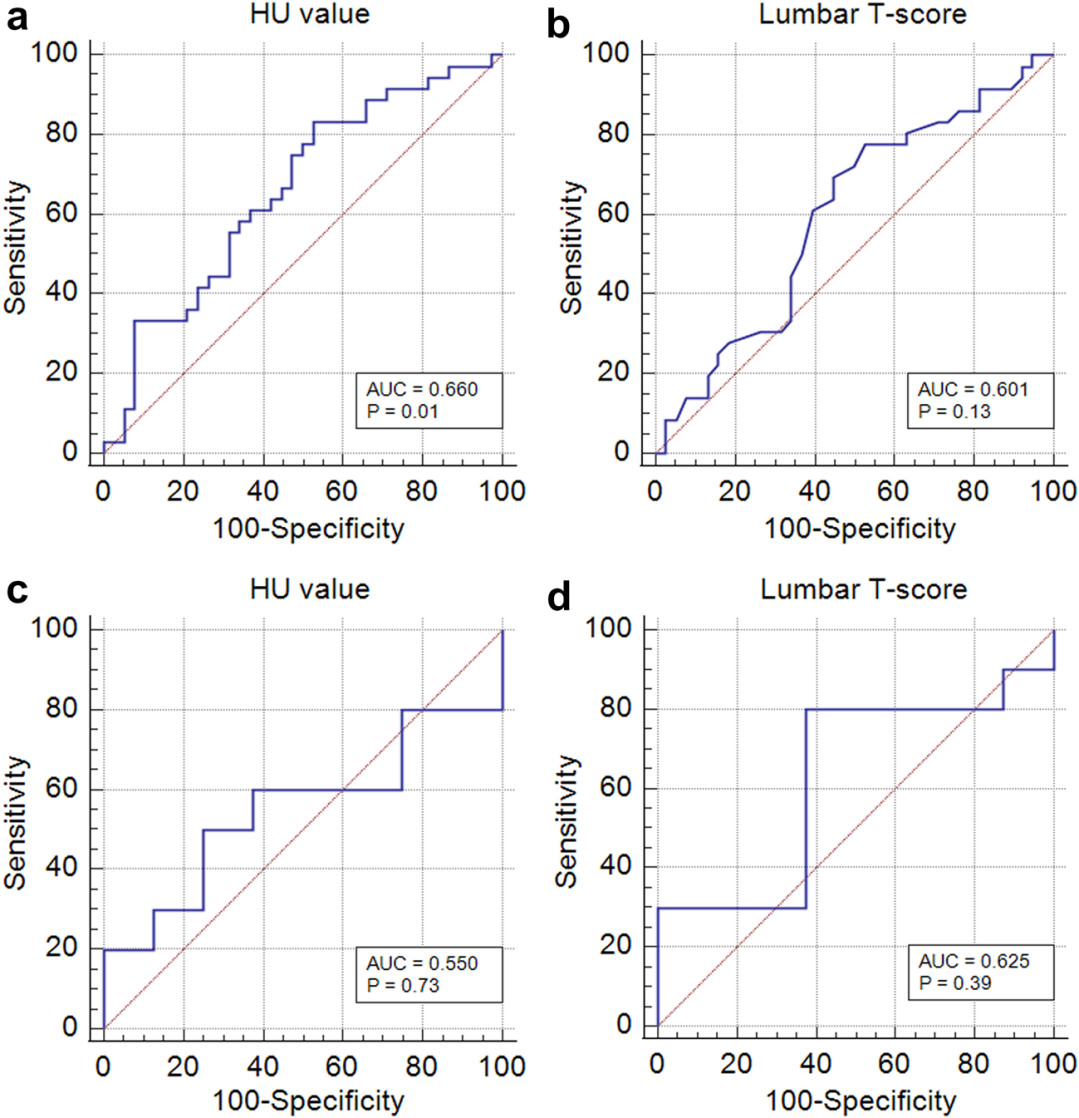
Receiver operating characteristic curves of HU values and lumbar T-scores according to the level of upper instrumented vertebra (UIV): lower thoracic UIV (**a**,**b**) and upper thoracic UIV (**c**,**d**). HU, Hounsfield unit; AUC, area under the receiver operating characteristic curve.

**Figure 4:**
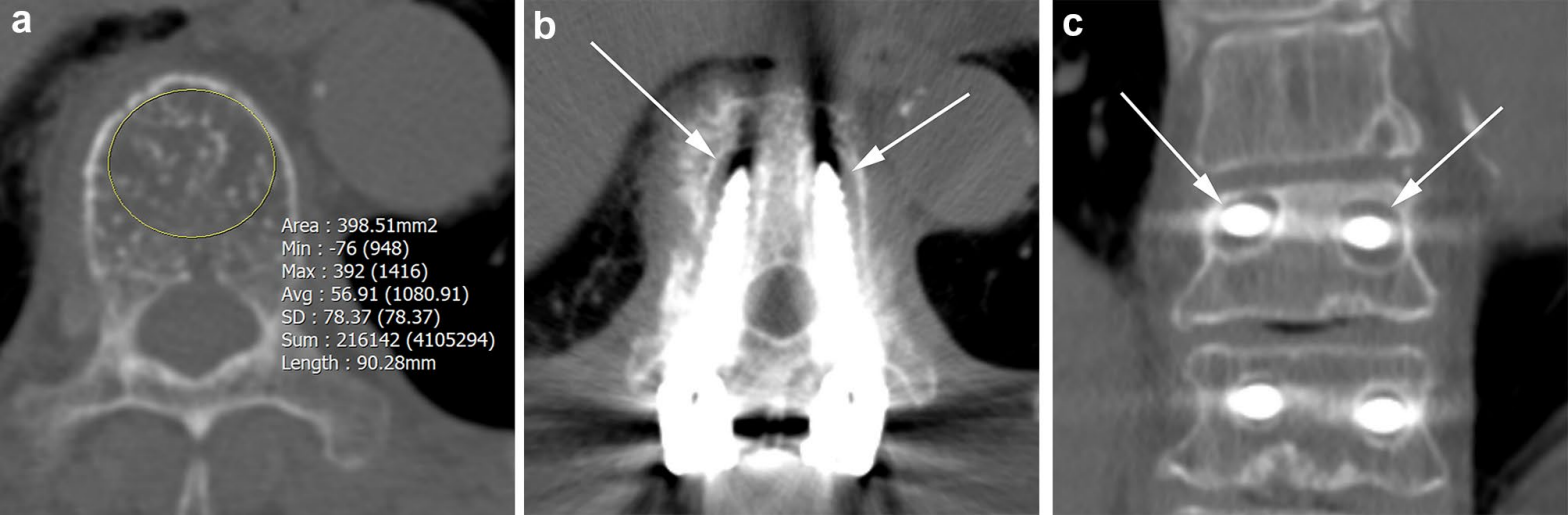
A 76-year-old woman who underwent spinal fusion surgery from T10 to S1. In the axial plane of the preoperative CT scan (**a**), the Hounsfield unit value of the upper instrumented vertebral body (T10) was 56.91, which was lower than the cutoff value. Postoperative CT images performed 1 year after surgery revealed bilateral pedicle screw loosening in both the axial (**b**) and coronal (**c**) planes (arrows). *min* minimum, *max* maximum, *avg* average, *SD* standard deviation.

**Figure 5:**
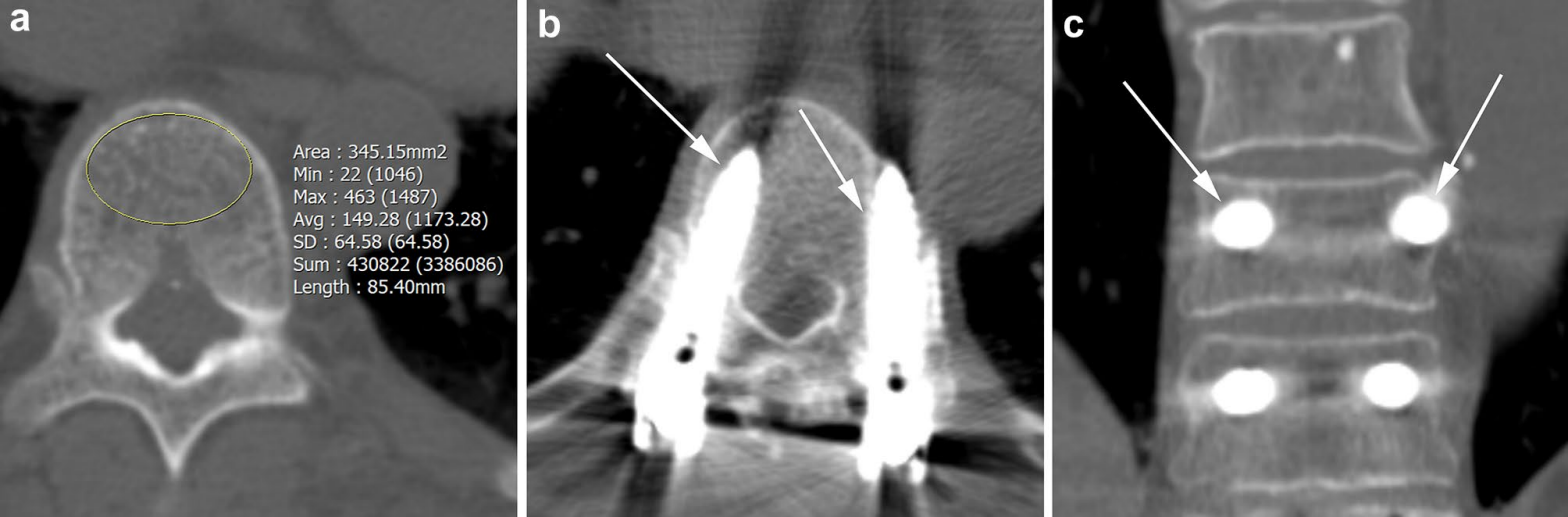
A 77-year-old woman who underwent spinal fusion surgery from T10 to S1. In the axial plane of the preoperative CT scan (**a**), the Hounsfield unit value of the upper instrumented vertebral body (T10) was 149.28, which was higher than the cutoff value. Postoperative CT performed 2 years and 7 months after surgery did not show screw loosening in the axial (**b**) or coronal (**c**) planes (arrows). *min* minimum,*max* maximum, *avg* average, *SD* standard deviation.

The AUC for the prediction of screw loosening was 0.610 (*p* = 0.07; 95% confidence interval [CI] 0.503–0.710) for HU values and 0.557 (*p* = 0.35; 95% CI 0.450–0.661) for T-scores. In patients with UIVs located at the lower thoracic level, the AUC was 0.660 (*p* = 0.01; 95% CI 0.541–0.766) for HU values and 0.601 (*p* = 0.13, 95% CI 0.480–0.713) for T-scores (Fig. 3a,b). The optimal cutoff HU value for the lower thoracic level was 126.3. In patients with UIVs at the upper thoracic level, the AUC was 0.550 (*p* = 0.73, 95% CI 0.303–0.760) for HU values and 0.625 (*p* = 0.39; 95% CI 0.370–0.837) for T-scores (Fig. 3c,d). The differences in AUCs between the HU values and T-scores at the upper and lower levels, upper thoracic level, or lower thoracic levels were not statistically significant (*p* = 0.36, 0.54, and 0.33, respectively). Representative cases of similar ages and surgical levels are shown in Figs. 4 and 5.

### Sensitivity, specificity, positive predictive value, negative predictive value, and accuracy

HU values were dichotomized with a cutoff value of 126, rounded down from 126.3, based on the AUC curve analysis. T-scores were dichotomized into an osteoporosis group (T-score ≤ − 2.5) and a non-osteoporosis group (T-score > − 2.5). In patients with UIVs at the lower thoracic level, the HU values showed superior PPV, NPV and accuracy compared to the T-scores in predicting screw loosening. A similar trend was observed in patients with UIVs at the upper thoracic level, although the number of samples was small. These results are summarized in Table 3, along with the numerators and denominators of the percentages.

**Table 3:** Sensitivity, specificity, PPV, NPV, and accuracy of HU value and T-score for prediction of screw loosening.

Parameter	Sensitivity (%)	Specificity (%)	PPV (%)	NPV (%)	Accuracy (%)
**All patients**					
HU value^†^	71.7 (33/46)	47.8 (22/46)	57.9 (33/57)	62.9 (22/35)	59.8 (55/92)
T-score^‡^	28.3 (13/46)	71.7 (33/46)	50.0 (13/26)	50.0 (33/66)	50.0 (46/92)
**Lower thoracic UIV**					
HU value	80.6 (29/36)	47.4 (18/38)	59.2 (29/49)	72.0 (18/25)	63.5 (47/74)
T-score	30.6 (11/36)	73.7 (28/38)	52.4 (11/21)	52.8 (28/53)	52.7 (39/74)
**Upper thoracic UIV**					
HU value	40.0 (4/10)	50.0 (4/8)	50.0 (4/8)	40.0 (4/10)	44.4 (8/18)
T-score	20.0 (2/10)	62.5 (5/8)	40.0 (2/5)	38.5 (5/13)	38.9 (7/18)

*PPV* positive predictive value, *NPV* negative predictive value, *HU* Hounsfield unit, *UIV* upper instrumented vertebra.

^†^Dichotomized with a cutoff value of 126.

^‡^Dichotomized with a cutoff value of − 2.5.

## Discussion

In this study, patients with screw loosening tended to have lower HU values than those in the control group, although the difference was not statistically significant (*p* = 0.07). At the lower thoracic level (T9–T12) of the UIV, patients with screw loosening had significantly lower HU values than the control group (99.30 vs. 126.32, *p* = 0.02). The HU values demonstrated a statistically significant predictive performance for screw loosening in the receiver operating characteristic curve analysis, and the optimal cutoff value was 126. Using this cutoff, the HU values showed better PPV, NPV, and accuracy compared to the T-scores.

Long-segment instrumentation is a well-known risk factor for pedicle screw loosening according to previous studies^5,15^. In the lumbar spine, the T-score obtained using DEXA is most commonly used for risk assessment. However, DEXA can provide false-negative results. Moreover, it is not generally accepted as a reliable test method for BMD in the thoracic spine. It has consistently been reported that the measurement of HU values on CT scans can be an alternative method of BMD assessment, as it demonstrates good correlation with DEXA results when evaluating osteoporosis^11,16,17^. In the lumbar region, a few studies have shown a correlation between lower HU values and screw loosening^13,14^. According to a previous study by Duan et al.^18^, lower HU values were associated with proximal junctional kyphosis in patients who underwent long-segmental fusions involving the lower thoracic spine. Our results also demonstrated a difference in HU values between the loosening and control groups at the lower thoracic level. The HU value appears to have the potential to predict pedicle screw loosening in the thoracic spine, where DEXA is not feasible. Furthermore, the cost and radiation exposure can be reduced by utilizing preoperative CT without the need for additional studies.

Duan et al.^18^ suggested a cutoff value of 104 HU for predicting the risk of proximal junctional kyphosis in the lower thoracic spine (T9–T12). A higher cutoff value of 126 HU was derived from our study. This is probably because of differences in the observed variables. Our study included patients with pedicle screw loosening in their UIVs, with or without kyphosis. Given that pedicle screw loosening can lead to proximal junctional kyphosis, it can be assumed that our study included patients with a broader spectrum of proximal junctional failure. In this context, a slightly higher cutoff value is reasonable. The cutoff value in our study was generally consistent with the cutoff value of approximately 120 HU reported as a risk factor for pedicle screw loosening by previous lumbar spine studies^13,14^.

There have been reports that the frequency of postoperative complications may depend on the UIV level^19,20^. However, the role of the UIV level in proximal junctional failure has been inconsistently reported^21,22^. According to previous risk factor analyses, vertebral body fracture was the most common cause of proximal junctional failure at the lower thoracic level, whereas soft tissue failure or subluxation was the main cause at the upper thoracic level^23,24^. In our study, lower HU values were associated with a higher rate of screw loosening at the lower thoracic level, but not at the upper thoracic level. This implies that vertebral bone quality is more closely related to screw loosening and proximal junctional failure at the lower thoracic level than that at the upper thoracic level. Further studies are warranted to investigate the difference in the mechanism of proximal junctional failure between the upper and lower thoracic spine.

This study had a few limitations. First, it was a single-center retrospective study with a relatively small sample size. In particular, the number of patients with upper-thoracic level instrumentation was small. A larger study seems necessary to advance the understanding of the effect of UIV on the relationship between HU values and pedicle screw loosening. Second, regions of interest drawn manually by only one observer were used for the HU measurements. However, this does not appear to have significantly impaired the reproducibility of the study since the interobserver agreement of manual vertebral bone attenuation measurement was described as good to excellent in a previous study^25^.

In conclusion, when the UIV was located at the lower thoracic level, patients with pedicle screw loosening had lower HU values of the vertebral body than those without pedicle screw loosening. HU value is a potential biomarker for predicting pedicle screw loosening at the lower thoracic level.

## Data Availability

The datasets generated during and/or analysed during the current study are available from the corresponding author on reasonable request.
